# Computer-Delivered and Web-Based Interventions to Improve Depression, Anxiety, and Psychological Well-Being of University Students: A Systematic Review and Meta-Analysis

**DOI:** 10.2196/jmir.3142

**Published:** 2014-05-16

**Authors:** E Bethan Davies, Richard Morriss, Cris Glazebrook

**Affiliations:** ^1^Division of Psychiatry and Applied Psychology, School of MedicineInstitute of Mental HealthThe University of NottinghamNottinghamUnited Kingdom

**Keywords:** systematic review, meta-analysis, intervention, universities, students, mental health, depression, anxiety, health promotion

## Abstract

**Background:**

Depression and anxiety are common mental health difficulties experienced by university students and can impair academic and social functioning. Students are limited in seeking help from professionals. As university students are highly connected to digital technologies, Web-based and computer-delivered interventions could be used to improve students’ mental health. The effectiveness of these intervention types requires investigation to identify whether these are viable prevention strategies for university students.

**Objective:**

The intent of the study was to systematically review and analyze trials of Web-based and computer-delivered interventions to improve depression, anxiety, psychological distress, and stress in university students.

**Methods:**

Several databases were searched using keywords relating to higher education students, mental health, and eHealth interventions. The eligibility criteria for studies included in the review were: (1) the study aimed to improve symptoms relating to depression, anxiety, psychological distress, and stress, (2) the study involved computer-delivered or Web-based interventions accessed via computer, laptop, or tablet, (3) the study was a randomized controlled trial, and (4) the study was trialed on higher education students. Trials were reviewed and outcome data analyzed through random effects meta-analyses for each outcome and each type of trial arm comparison. Cochrane Collaboration risk of bias tool was used to assess study quality.

**Results:**

A total of 17 trials were identified, in which seven were the same three interventions on separate samples; 14 reported sufficient information for meta-analysis. The majority (n=13) were website-delivered and nine interventions were based on cognitive behavioral therapy (CBT). A total of 1795 participants were randomized and 1480 analyzed. Risk of bias was considered moderate, as many publications did not sufficiently report their methods and seven explicitly conducted completers’ analyses. In comparison to the inactive control, sensitivity meta-analyses supported intervention in improving anxiety (pooled standardized mean difference [SMD] −0.56; 95% CI −0.77 to −0.35, *P*<.001), depression (pooled SMD −0.43; 95% CI −0.63 to −0.22, *P*<.001), and stress (pooled SMD −0.73; 95% CI −1.27 to −0.19, *P*=.008). In comparison to active controls, sensitivity analyses did not support either condition for anxiety (pooled SMD −0.18; 95% CI −0.98 to 0.62, *P*=.66) or depression (pooled SMD −0.28; 95% CI −0.75 to −0.20, *P*=.25). In contrast to a comparison intervention, neither condition was supported in sensitivity analyses for anxiety (pooled SMD −0.10; 95% CI −0.39 to 0.18, *P*=.48) or depression (pooled SMD −0.33; 95% CI −0.43 to 1.09, *P*=.40).

**Conclusions:**

The findings suggest Web-based and computer-delivered interventions can be effective in improving students’ depression, anxiety, and stress outcomes when compared to inactive controls, but some caution is needed when compared to other trial arms and methodological issues were noticeable. Interventions need to be trialed on more heterogeneous student samples and would benefit from user evaluation. Future trials should address methodological considerations to improve reporting of trial quality and address post-intervention skewed data.

## Introduction

Depression and anxiety are common mental health problems experienced by university students [1]. A recent review reported a 30.6% mean prevalence rate of depression in students [2] and a cross-sectional survey reported 17.3% prevalence of clinically-significant psychiatric caseness in a UK student sample [3]. Being in higher education is associated with many stressors and transitional events, and students fall within the age range when common mental health problems are at their developmental peak [4]. Of students who screened below the threshold for anxiety and depression at entry to university, 9% were above the threshold for depression and 20% for anxiety 18 months into their course [5]. Depression and anxiety can impair students’ academic performance and social functioning, cause significant burden at university, and potentially affect their future career opportunities [4,6,7]. Students’ help-seeking behavior for their mental health difficulties is limited, with many not contacting relevant professional services [8]. Young people do not seek out help for several reasons, including personal preferences for self-reliance in managing their mental health [9].

Computer-delivered and Internet-enabled interventions have been increasingly trialed in recent years [10]. Programming technology means interventions can be delivered using a range of multimedia formats and interactive features to engage users and facilitate intervention efficacy [11]. Computer- and Internet-delivered interventions hold many advantages; they can be tailored to student needs, accessed anonymously, and provide a more comfortable private environment to access sensitive information [12]. Online interventions can be a form of outreach to individuals who may not access traditional face-to-face services [13]. Evidence-based psychotherapies have been effectively adapted for Internet-based delivery, with much evidence supporting computer-delivered cognitive behavioral therapy (CCBT) in improving depression and/or anxiety outcomes [14-17]. The Internet is an essential tool for higher education and thus highly accessible to students [12,18]. Students also use the Internet for health-related purposes; over a third of students stated that information found via the Internet had a significant effect on their own health self-care [18]. Given that students may not seek out professional help for their mental health, computerized technologies could provide access to self-help. Students may have favorable preferences toward self-help due to their increasing independence and ability to be self-reliant during their transition to young adulthood [19]. Over half of students in an Australian sample who screened for high psychological distress reported strong intentions in using an online program for student well-being [12]. As Internet-based interventions have been cited as an approach that may be particularly engaging and useful for higher education students given their limited help-seeking behavior [12,20-22], there is a need to identify and synthesize the evidence from these types of interventions for improving common mental health difficulties in higher education populations. Several UK universities appear to offer online counselling to their students, but students still have to engage in help-seeking behavior to access these services and may have stigmatizing attitudes toward professional help [23]. Self-guided computer and Internet-based resources may help to avoid this stigma and be in line with preferences for self-reliance. The recent systematic review by Farrer and colleagues [4] explored technology-based interventions trialed in higher education populations and has provided a comprehensive narrative appraisal of these trials. However, quantitative analysis was not conducted due to the variation of technologies employed in the studies. We hope to expand on this by focusing only on interventions delivered through websites and offline computer programs for improving mental health outcomes, and conducting meta-analysis to explore these outcomes. Analysis of this type of intervention in student populations has not been explored previously. The aim of this review is to explore whether computer-delivered and Web-based (ie, website-based) interventions are effective in improving depression, anxiety, and psychological well-being in higher education students.

## Methods

### Search Methodology and Identification of Trials

Nine electronic databases, including PsychINFO, CENTRAL, and PsychMed, were searched in March-April 2012; the search was repeated in June 2013 to ensure the search was as current as possible. Search terms (Multimedia Appendix 1) were developed through literature review and related to Internet- and computer-delivered interventions, mental health, and higher education. Several publisher websites, published reviews, and intervention studies were hand-searched. There was no restriction in year or language of publications. Studies met the following eligibility criteria:

1. The interventions had to aim to improve psychological distress, stress, depressive, or anxiety symptomology, and had administered valid and reliable measure(s) reflecting this symptomology. Interventions that also addressed general aspects of psychological well-being (eg, sleep) and included a primary mental health outcome were also included.
2. The intervention was delivered via a website or offline computer program and accessed via computer, laptop, or other technological device (eg, tablet). These technological mediums were used as a medium for delivering the intervention. Human support was included in the review providing it was delivered by laypersons or non-health care professionals and was a complementary component of intervention.
3. The study was published in a peer-reviewed journal.
4. The intervention was trialed through randomized controlled trial (RCT) design. Trial arms need to consist of an experimental condition and an inactive control (ie, no-treatment or wait list control) condition and/or an active control and/or comparison intervention. Active control was defined as participants who received materials designed to mimic the time and attention received by participants assigned to the intervention. Active controls were not designed to produce the same changes upon outcomes as expected in the intervention.
5. The intervention was trialed on undergraduate and/or postgraduate students in higher education institutions [HEIs]. HEIs were tertiary educational institutions, such as universities and colleges.

Secondary outcomes of interest were help-seeking behavior, mental health service utilization, diagnosis of mental disorder, and participant attrition. Interventions were excluded if there was face-to-face human support adjunct to intervention, they were not Web-based or offline computer programs, they were online support groups, or were mobile or tablet applications. Interventions that utilized computers/Internet to facilitate communication (eg, email, online counselling) between health professionals and users were also excluded as we wanted to explore whether computer-delivered and Web-based interventions were comparable to traditional therapies (eg, face-to-face CBT) and had any effects on mental health outcomes in comparison to receiving no treatment. Mobile applications (“apps”) were also excluded as, at the time of conducting the search, it was felt these were relatively new mediums in terms of therapeutic interventions and appeared more likely to be used as a device to display information in the same way as a DVD/video. Online interventions for eating disorders and alcohol/substance use were not included as these have been previously reviewed in students [24,25]. Publications were excluded if they focused on mediating effects upon outcome measures only within experimental groups, or if both the intervention and active control/comparison intervention received the same intervention materials and there was no inactive control condition.

**Figure 1:**
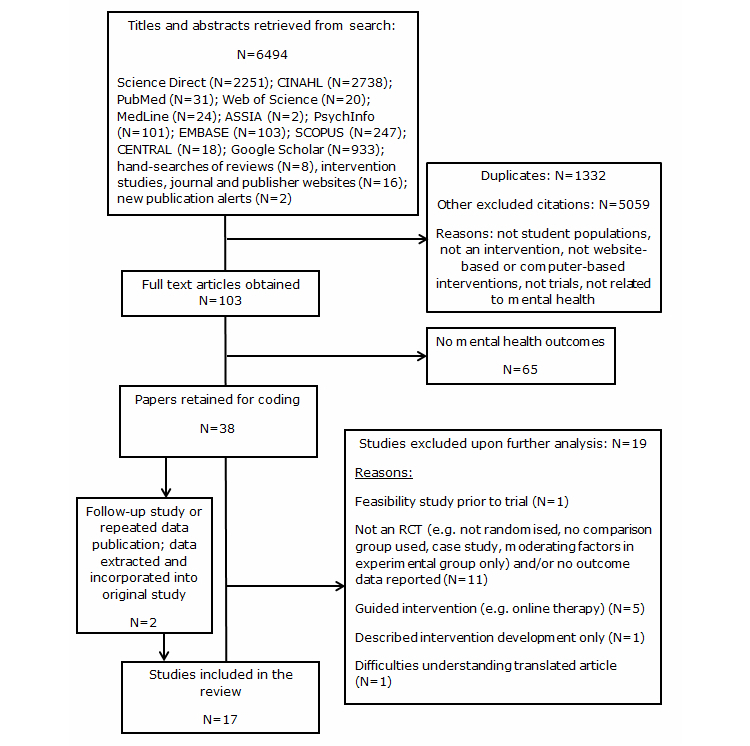
PRISMA flowchart outlining process for systematic review/meta-analysis.

A total of 6494 titles and abstracts were retrieved from the search and screened by EBD to address their inclusion eligibility. Reference lists of relevant reviews were also searched. The updated search resulted in inclusion of some additional studies that were not published at the time of the first search. The full text of 103 articles was obtained for further analysis and coding. Of these, 38 addressed the targeted mental health criteria and 19 were excluded as they did not meet eligibility criteria or presented translation difficulties [26] (see Multimedia Appendix 2 for further description). A total of 19 articles met inclusion criteria, which included one follow-up to an included study [27] and two publications reporting the same trial [28,29]; data from both were extracted and collapsed into the original studies, resulting in 17 citations. Figure 1 outlines the search process (also see Multimedia Appendix 3).

### Data Extraction and Assessment

Data extraction was performed by EBD using a template based on the Cochrane Review template [30] and the CONSORT checklist for reporting eHealth interventions [31]. Authors were contacted if necessary to clarify information. Data regarding post-intervention means and standard deviations from relevant mental health outcome measures and information about participant attrition were extracted from the included studies and entered into Review Manager (“RevMan”) software [32].

Interventions were classified by their type of prevention [33]: “universal interventions” target a whole population regardless of individual risk and do not involve screening; “selective interventions” select individuals at some risk of a mental health disorder but without screening of mental health symptomology; “indicated interventions” target those who screen for some level of mental health symptomology but do not have a diagnosis; and “treatment interventions” are delivered to individuals with a diagnosed mental disorder [4]. For this review, “selective” and “indicated” interventions were collapsed into one category as it can be difficult to decipher whether interventions discretely fit into one category.

The level of human support provided to participants was coded using categories used previously [4,34]. Only three categories were used as we did not aim to explore interventions that involved extensive contact time between participants and a human contact. The three categories were: (1) no-contact intervention (no human face-to-face or verbal contact for any aspect of study; email contact only with participants), (2) self-administered intervention (human contact for administration of measures only), and (3) semi-guided intervention (human contact ≤90 minutes for prompts or reminders, guidance on intervention use, and/or support in completing intervention).

The Cochrane Collaboration risk of bias tool [35] was used to assess trial quality. The tool provides a checklist to aid understanding of trial quality and does not calculate an overall quality score. The tool assesses study bias across five methodological domains: sequence generation, allocation concealment, blinding, incomplete outcome data, and selective reporting.

### Process for Meta-Analysis

Meta-analyses were planned to explore the effects of interventions upon depression, anxiety, stress, and psychological distress related outcomes. These outcomes were analyzed in three subgroups: (1) comparing intervention to inactive control, (2) comparing intervention to active control, and (3) intervention compared to comparison intervention. If trials conducted three or more trial arms, the trial arms were separated corresponding to the three comparison analyses. In studies using two or more active control or comparison intervention conditions, the least active control was entered into analysis. Secondary analyses were conducted to explore year of publication and use of participant incentives upon outcomes, as well as exploring rates of attrition between trial arms. Continuous data on clinical outcomes are often not normally distributed and extracted data were explored for normality via presence of skew. This is done by multiplying the standard deviation by two; if the mean is smaller than this number, it suggests the data is skewed [36]. RevMan was used for calculating effect sizes and conducting meta-analyses. Standard errors were transformed into standard deviations by multiplying the standard error by the square root of the sample size. If insufficient outcome data were reported for extraction, those studies were not included in meta-analysis. If studies reported more than one type of outcome measure for specific outcomes of interest, the measure most aligned to DSM-IV criteria for depressive and anxiety disorders was selected for analysis. The Standardized Mean Difference (SMD) is a version of effect size typically calculated in reviews and is expressed as Hedges’ *g*. SMDs were calculated for each included study by subtracting the post-intervention mean of the intervention condition from the post-intervention mean of the comparison condition, and dividing this by the pooled standard deviation from both conditions [37]. Use of SMD allows for comparisons across included studies where they used different psychometric measures to assess the same outcomes [38]. Inferences of Hedges’ *g* can be made using Cohen’s *d* conventions as small (0.2), medium (0.5), and large (0.8) [39].

We anticipated included studies would be heterogeneous due to the different types of preventative intervention and so would differ on the baseline symptomology of participants. To help account for the expected heterogeneity, Random Effects Models (RAM) with 95% confidence intervals (CI) were applied throughout analysis. RAM assumes that included studies are trialed on different populations and so are calculating different intervention effects [38,40]. The *I* ^*2*^statistic was calculated to explore heterogeneity and is expressed as a percentage indicating its degree: 25% indicates low heterogeneity, 50% suggests moderate, and 75% is a threshold marker for high heterogeneity [41]. The *Q* statistic was also calculated and provides the statistical significance of heterogeneity.

## Results

### Intervention Characteristics

The search yielded 17 studies. The symptomology measured within trials were depression [28,42-52], anxiety [28,42-48,50], stress [46,53-55], psychological distress [50,54,56], social anxiety [52], and examination anxiety [57]. Some interventions focused on general psychological well-being: improving relationship functioning [43,44], decreasing elevated levels of perfectionism [28,42], increasing students’ use of mindfulness [54], improving international students’ social support, acculturation, and hardiness [56], and increasing use of lucid dreaming to help alleviate depression [51]. Of the studies, seven trials were of three interventions conducted on separate samples; therefore, there are 14 distinct interventions for review. Multimedia Appendix 4 provides a summary of included interventions.

A total of 11 trials were selective or indicated interventions, where participants were included if they were screened for specific aspects of mental health symptomology or other psychological factors [28,42,45-50,53,55,57]. Inclusion criteria included: elevated perfectionism [28,42], elevated stress [53,55], minimal/mild symptoms of depression and anxiety [45,50], low/moderate psychological distress [47], elevated anxiety sensitivity [48], elevated psychological distress [49], self-reported examination anxiety [57], and mild/moderate levels of depression, anxiety, or stress [46]. Five trials were universal, in which mental symptomology were not explicit inclusion criteria; participants had to be in ≥4 month long romantic relationships [43,44], be Indian international students [56], have no lucid dreaming experience [51], or have access to an Internet-connected computer [54]. One intervention was treatment as participants met DSM-IV diagnostic criteria for social anxiety [52]. It is difficult to decipher whether some included trials discretely fitted the selective or indicated type. Some studies recruited participants with minimal symptomology or focused on other risk factors for depression and anxiety, such as elevated perfectionism [28,42].

Of the studies, 11 contained two trial arms [42,44-46,48,49,51,54-57], with five using three arms [28,43,47,52,53], and one study with four arms [50]. Five trials compared intervention to inactive (ie, no treatment or waitlist control) and either an active control [53] or comparison intervention [28,47,50,52], five trialed the intervention to an active control [44,45,55-57], six trialed against inactive control [42,46,48,49,51,54], and one compared intervention to a comparison intervention and active control [43]. Further, 13 studies [28,42-50,52,55,57] trialed interventions based on CBT; this included seven studies in which three interventions were trialed on separate samples [28,42-44,47,49,50]. Other interventions were based on mindfulness [54], stress management theory and cognitive learning theory [53], and lucid dreaming [51].

### Location and Delivery of Intervention

The majority of interventions were delivered via a website or university intranet (n=13) with four being offline computer programs [43-45,55]. Five trials were delivered at a study site, eg, researcher-monitored computer lab [43-45,47,50,55], while participants in six Internet-based interventions accessed the intervention in their own location [48,49,52,53,56,57]. A total of 14 trials had interventions with an modular/sectional format [28,42-48,50,52,54-57] ranging from three [56] to 13 modules [28,42]. The other trials coupled module-based (“MoodGym”) and psycho-educational (“BluePages”) websites [49], provided biweekly instruction via a website [51], and included a psycho-educational stress management website [53]. The intervention delivery period ranged from 2 [53,54] to 12 weeks [42], with median length of 6 weeks. All studies reported short-term outcomes (≤12 weeks) with measures usually administered at the end of the trial. Five reported additional follow-up at 6 months [46,48,53], 10 months [44], and 1 year post-baseline [52]. Four Web-based interventions stated how much time was required to spend accessing the intervention: at least four 20-minute periods over 2 weeks [53]; 1 hour per week over 3 weeks [47]; 30 minutes per week over 6 weeks [57]; and 5-7 days for each module [48]. The four computer-delivered interventions took between 30 to 120 minutes [43-45,55] to complete and were supplemented by weekly standardized emails.

### Use of Human Support in Interventions

Seven trials were classified as self-administered [28,42,48,51-53,57], with nine being semi-guided [43-47,50,54-56]. Participants in one trial received no reminders but it was unsure if there was face-to-face/verbal contact between researchers and participants [49]. For semi-guided interventions, six trials involved sending standardized emails periodically to encourage participants to complete the intervention [54,56], or to remind participants about the principles learned in the computer-based intervention [43-45,55]. Chiauzzi [53] sent reminder emails only if participants were not accessing the intervention for the required duration. Two trials featured weekly telephone or email-based support from a “program coach” [46] or from the researchers [55] to help participants complete the intervention or to prompt skills practice. Six trials [43-45,47,50,55] were carried out at a study site where a researcher was present to provide support and aid participants’ familiarity with the intervention. One intervention involved peer interaction via online forum [56]. Three offline computer-delivered interventions involved a single session of participant-computer interaction, supplemented with hard copies of the presented material [43,44] or worksheets to complete after experiencing a stressful encounter [45]. The additional computer-delivered intervention was accessed weekly over 6 weeks and was supplemented with hard copies and a practice version of the intervention on a USB flash drive for off-site personal access [55].

### Participant Characteristics

A total of 1795 participants consented and were randomized to a trial arm. Sample sizes ranged from 38 [50] to 240 [53]. Four trials had samples of ≥150 participants [45,49,51,53]. Overall, 1480 were explicitly included in analyses. Seven studies explicitly stated analysis was conducted on participants who completed pre-post intervention measures [28,42,45,48,55-57], while eight studies conducted intention-to-treat [ITT] analyses [44,46,47,49,50,52-54]. ITT was conducted through using maximum likelihood estimation [44,46], mixed-models repeated measures [49], mixed-models analysis [53], and by carrying last observation forward [52,54]. One reported separate ITT, completers, and compliers analyses [49]. Uncertainty about types of analysis was present in two publications [43,51]; 12 publications provided information regarding participant dropouts/withdrawals: dropout rates ranged from 7.2% [28] to 44.2% [54]. Five provided some reason for withdrawal; this included not receiving response to researcher’s contact [44], personal time constraints [42,48,52], personal reasons [42], concerns about intervention efficacy [52], participants felt better after receiving some intervention modules [52], and participant requested face-to-face therapy instead [49].

The 10 studies describing their sample’s age range included participants ranging from 17 to 51 years. In 15 trials, participants’ mean age ranged from 18.37 to 28.2 years; their mean age from these was 22.6 years. All studies recruited males and females, with females being the majority in 15 studies. Gender balance varied from 50% [55] to 88.46% [54] of the sample being female. A total of 10 trials were conducted on undergraduate populations [28,42-45,47,48,50,51,53], five on both undergraduates and postgraduates [46,49,52,54,57], and two on postgraduates only [55,56]. Psychology students were overrepresented in the undergraduate studies with seven recruiting psychology undergraduates only [28,42-45,48,50] and another recruiting psychology and health sciences students [47]. Likewise, seven trials reported use of course or financial credit for participation [42-45,47,50,51,55]. The majority of trials (n=7) were conducted in HEIs in the United States [43-45,51,53,56], with three trials in Canada [28,42,46] and Australia [47,48,50], two in the United Kingdom [54,57], one in Spain [52] and Norway [49]. Further, 13 trials were conducted within one HEI [28,42-45,47,48,50,51,54-57]; the others recruited students at two [49,52], three [46], and six [53] HEIs.

### Multimedia Use and Interactivity of Interventions

Limited information was provided regarding multimedia and interactivity. Text was presented in all interventions, with the use of images/graphics also reported [43,44,47-49,53,56]. Animation, music, and audio voiceovers were used in the examination anxiety intervention [57], and the social anxiety intervention utilized streaming of online videos to expose participants to an anxiety-inducing situation [52]. MoodGym [47,49,50] included interactive activities and an online workbook. Recently published studies appeared to provide more information on the presentation and interactivity of intervention content. Day [46] reported each module was presented using a range of videos, audio, pictures, and interactive activities. Mindfulness was taught through text and videos, and participants were able to choose to listen to either a male- or female-delivered 10-minute audio of meditation instruction [54]. SMART-OP [55] incorporated animation, videos, and text to create a tailored user experience, as well as using game-like interactive tasks.

### Outcome Measures Used

A small number of established valid and reliable measures were used to primarily measure depression, anxiety, psychological distress, and stress outcomes (see Table 1). Stress is an important psychological well-being outcome given that students are faced with several stressors during their studies and elevated stress can increase the risk of developing mental health difficulties [58]. All trials administered self-report measures to participants, either through hard copy or through online administration. One study administered the Trier Social Stress Test and measured associated physiological stress responses [55].

**Table 1:** Outcome measures used for assessment of depression, anxiety, psychological distress, and stress in the included studies.

Author	Anxiety					Depression			Psychological distress		Stress	
	ASI^a^	BAI^b^	DASS-21^c^	SAD^d^	TAI^e^	BDI^f^	CES-D^g^	DASS-21	K10^h^	PHQ-4^i^	PSS^j^	DASS-21
Arpin-Cribbie 2012	✓	✓					✓				✓	
Botella 2010				✓		✓						
Braithwaite 2007		✓				✓						
Braithwaite 2009		✓				✓						
Cavanagh 2013										✓	✓	
Chiauzzi 2008											✓	
Cukrowicz 2007		✓					✓					
Day 2013	✓		✓					✓				✓
Ellis 2011			✓					✓	✓			✓^k^
Kanekar 2010									✓^l^			
Kenardy 2003	✓						✓					
Lintvedt 2011							✓					
Orbach 2007					✓							
Radhu 2012		✓					✓				✓	
Rose 2013											✓	
Sethi 2010			✓					✓	✓			✓^k^
Taitz 2011						✓						

^a^ASI: Anxiety Sensitivity Inventory

^b^BAI: Beck Anxiety Inventory

^c^DASS-21: Depression Anxiety and Stress Scale – 21 item version

^d^SAD: Social Avoidance and Distress scale

^e^TAI: Test Anxiety Inventory

^f^BDI: Beck Depression Inventory

^g^CES-D: Center for Epidemiologic Studies Depression Scale

^h^K10: Kessler Distress Scale – 10 item version

^i^PHQ-4: Patient Health Questionnaire – 4 item version

^j^PSS: Perceived Stress Scale

^k^Data from stress subscale of DASS-21 was not reported in the published article.

^l^Shorter version of scale used to analyze data collected on K10.

### Questionnaire Response Burden

Response burden reflects the amount of strain put on an individual to complete measures; factors influencing burden include length and intensity of measures and concentration required to complete them [59]. Response burden is a factor to consider in trials as participants typically complete a battery of measures at baseline and post-intervention, and potentially at more time-points during trials. Too many questions may increase burden and result in greater attrition or lower response rates [59]. We calculated the number of questions participants completed by reviewing the measures within included publications and totaling the approximated number of items within administered measures. It was estimated the measurement battery ranged from 25 [46] to 225 questions [42]. The estimated median number of questions presented to participants was 75 items.

### Participant Satisfaction/Evaluation With Intervention

Eight studies administered a form of participant evaluation [46-49,53-55,57]. Included interventions were reported to be highly useable [55], satisfactory [53], credible [48], and to be moderately-to-highly useful and helpful [46,47,49,54,57]. Cavanagh [54] directly asked participants if they felt the intervention had been beneficial; the majority felt the mindfulness intervention had at least some personal benefit. Day’s intervention [46] underwent usability, efficiency, and acceptability testing by university students prior to being trialed [60].

### Risk of Bias in Included Studies

**Figure 2:**
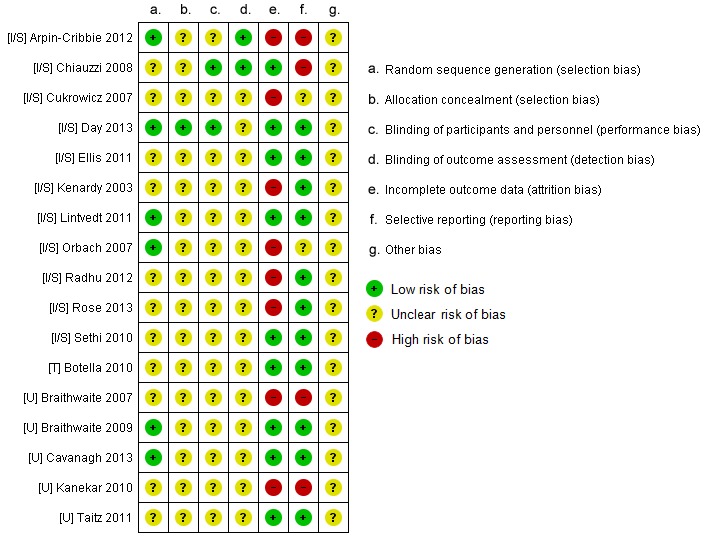
Breakdown of each type of risk of bias identified in the included studies.

We believed the risk of bias in included studies to be moderate—this was mostly due to publications being unclear or providing insufficient details (see Figure 2). All participants were randomized but only six studies [28,43,46,49,54,57] described their randomization method: a random number table [28], a computer-generated randomization sequence [43,46,49,54], and through tossing a coin [57]. Two studies [43,56] did not explicitly state how many participants were in each condition. It is viable to blind those collecting and/or assessing outcome data, as blinding participants can be difficult given the type of controls [14,31]. One study stated single-blindedness of participants and provided post-intervention evaluation of researchers’ non-blindedness [53]; another reported single-blindedness of researcher collecting data [28]. Seven studies [28,42,45,48,55-57] explicitly did completers’ analyses—overall, 208 participants were not included in analysis. Outcome data from three studies could not be extracted due to not reporting participant numbers in each condition [43,56], not reporting SD/standard error data [43,53], and assessing outcome data using a shortened version of the measure [56]. Gender balance is an issue as the majority of trials had more female than male participants. Baseline symptomology is also a potential source of bias for the review, as it may have caused some difficulties comparing intervention effectiveness in improving mental health outcomes. Trials varied in the level of mental health-related symptomology they targeted at baseline; some only recruited participants with minimal symptoms, while others wanted those experiencing elevated symptoms.

### Distribution of the Reported Data

Six studies explicitly stated their data had been checked for violations of assumptions of normality [28,45,49,53,55,57]. Two studies transformed skewed data for analysis to approximate a normal distribution [53,55], while Orbach [57] used non-parametric tests for skewed data. None of the included studies appeared to provide alternative measures of central tendency. Overall, there were 10 studies that reported skewed post-intervention on at least one primary outcome measure of interest [28,42,44-47,49,51,52,54].

### Meta-Analysis for Anxiety, Depression, and Psychological Distress Outcomes

Outcome data relating to the mental health symptomology measures was not extracted from three studies due to insufficient data reporting [43,53,56]. Orbach’s trial [57] was excluded from meta-analysis for anxiety outcomes, as test anxiety is considered an “extreme” reaction to examinations and is distinct from commonly diagnosable anxiety disorders [57]. Data regarding participant attrition could be extracted from two of these studies [53,57]. All mental health outcomes were continuous and scale-based, and were extracted as endpoint average scores with lower scores indicating fewer symptoms. Within the presented analyses, negative SMD values support the intervention condition.

Three analyses exploring intervention compared to inactive control, intervention compared to active control, and intervention compared to comparison intervention were conducted and are reported separately. For each type of comparison, outcomes relating to depression, anxiety, psychological distress, and stress are separately reported. For each outcome within each comparison, analyses are presented twofold: non-skewed data were analyzed first, with a secondary sensitivity analysis conducted to analyze skewed and non-skewed data on each outcome. If skewed data were present in one trial arm but not in the other, it was included in sensitivity analysis. Findings within forest plots were subgrouped by the separate measures used to measure each outcome in addition to calculation of an overall pooled effect. On all presented forest plots (see Figures), the bracketed letter before author name indicates their type: [U] universal intervention, [I/S] indicated or selective intervention, and [T] treatment intervention.

### Web-Based or Computer-Delivered Intervention Compared to Inactive Control

**Figure 3:**
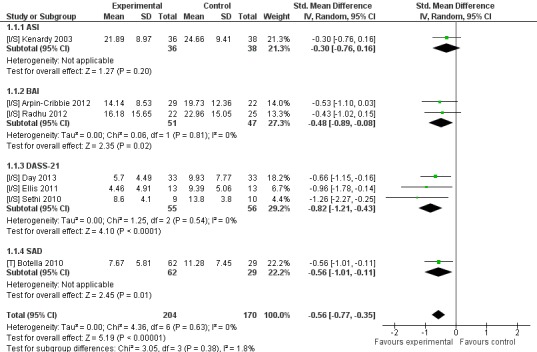
Sensitivity analysis of post-intervention anxiety outcomes for intervention compared to inactive controls.

Seven trials used this trial arm comparison to investigate effects of intervention upon anxiety outcomes. All trials were based on CBT and include four separate trials of two interventions [28,42,47,50]. Two trials reported non-skewed data—for these there was no difference between intervention and control for anxiety (n=93, 2 RCTs, pooled SMD −0.67, CI −1.59 to −0.25, *Z*=1.43, *P*=.15; *I* ^2^=66%, *P*=.09). Sensitivity analysis incorporated an additional five studies reporting skewed data. This analysis significantly favored the intervention (n=374, 7 RCTs, pooled SMD −0.56, CI −0.77 to −0.35, *Z*=5.19, *P*<.001; *I* ^2^=0%, *P*=.63; see Figure 3).

**Figure 4:**
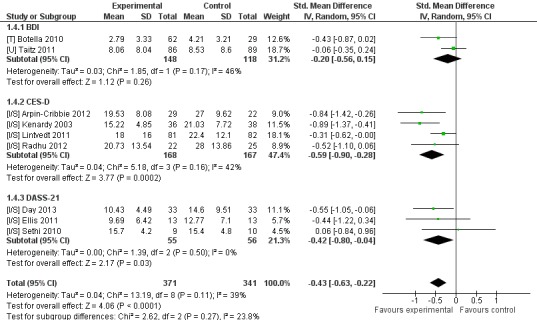
Sensitivity analysis of post-intervention depression outcomes for intervention compared to inactive controls.

Nine trials that compared intervention to inactive control reported depression outcomes. Eight trials had CBT-based interventions and included five separate trials of two interventions [28,42,47,49,50]. Three trials reported non-skewed outcome data and significantly favored intervention (n=144, 3 RCTs, pooled SMD −0.67, CI −1.15 to −0.20, *Z*=2.77, *P*=.006; *I* ^2^=43%, *P*=.17). A separate sensitivity analysis included an additional six studies reporting skewed data, with this analysis significantly favoring intervention (n=712, 9 RCTs, pooled SMD −0.43, CI −0.63 to −0.22, *Z*=4.06, *P*<.001; *I* ^2^=39%, *P*=.11; see Figure 4).

Two trials measured psychological distress, of which one reported skewed data [54]. Cochrane Collaboration guidelines suggest forest plots should not be produced for outcomes with single studies [61]; therefore, findings from the single non-skewed trial are presented in Multimedia Appendix 5. A sensitivity analysis was performed to include the additional study, which found no difference between intervention and control (n=123, 2 RCTs, SMD −1.39, 95% CI −3.79 to 1.02, *Z*=1.13, *P*=.26). Significantly high heterogeneity was present (*I* ^2^=92%, *P*<.001).

Three RCTs included an outcome measure of stress. For the two studies reporting non-skewed data, there was significant favorability for intervention (n=151, 2 RCTs, pooled SMD −0.44, CI −0.77 to −0.12, *Z*=2.68, *P*=.007; *I* ^2^=0%, *P*=.49). A separate sensitivity analysis included the additional skewed data, which significantly favored intervention (n=217, 3 RCTs, pooled SMD −0.73, CI −1.27 to −0.19, *Z*=2.64, *P*=.008). A significant high level of heterogeneity was present (*I* ^2^=72%, *P*=.03).

**Figure 5:**
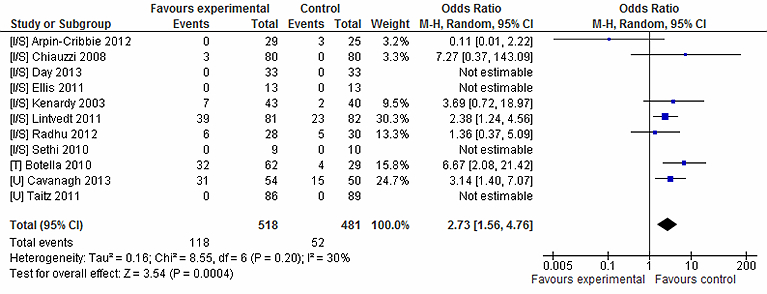
Attrition rates for intervention vs inactive control conditions.

Looking at attrition rates, participants were significantly more likely to leave the study early if they were randomly assigned to receive intervention (n=999, 11 RCTs, OR 2.73, CI 1.56-4.76, *Z*=3.54, *P*<.001; *I* ^2^=30%, *P*=.20; Figure 5). A total of 118 (22.7%) left the intervention condition early, compared to 52 (10.8%) in the inactive control condition.

### Web-Based or Computer-Delivered Intervention Compared to Active Control

There were seven trials that explicitly included an active control, but only three reported their outcome data relating to mental health outcomes of interest, or could not be included for reasons previously described. Data relating to attrition could be extracted from five of these trials. Two used the same active control in which participants viewed computer-based materials that provided descriptive information about depression and anxiety [44,45].

Two trials compared intervention to active control in investigating anxiety outcomes, both of which reported skewed data. Sensitivity analysis did not favor either intervention or active control (n=229, 2 RCTs, pooled SMD −0.18, CI −0.98 to 0.62, *Z*=0.45, *P*=.66). A high level of heterogeneity was reported (*I* ^2^=88%, *P*<.001). The same two trials also reported depression outcomes [44,45], which again were skewed. Sensitivity analysis did not support either condition (n=229, 2 RCTs, pooled SMD −0.28, CI −0.75 to 0.20, *Z*=1.14, *P*=.25; *I* ^2^=67%, *P*=.08).

**Figure 6:**
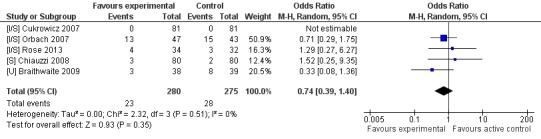
Attrition rates for intervention vs. active control conditions.

Only one trial assessed psychological distress within the intervention vs active control comparison [55]. It was not subject to analysis due to being the sole study (see Multimedia Appendix 5). There were no significant differences reported between attrition in the two arms (n=555, 5 RCTs, OR 0.74, CI 0.39-1.40, *Z*=0.93, *P*=.35; *I* ^2^=0%, *P*=.51; see Figure 6). A total of 23 (8.2%) participants left the intervention condition early, compared to 28 (10.1%) in the active controls.

### Web-Based or Computer-Delivered Intervention Compared to Comparison Intervention

**Figure 7:**
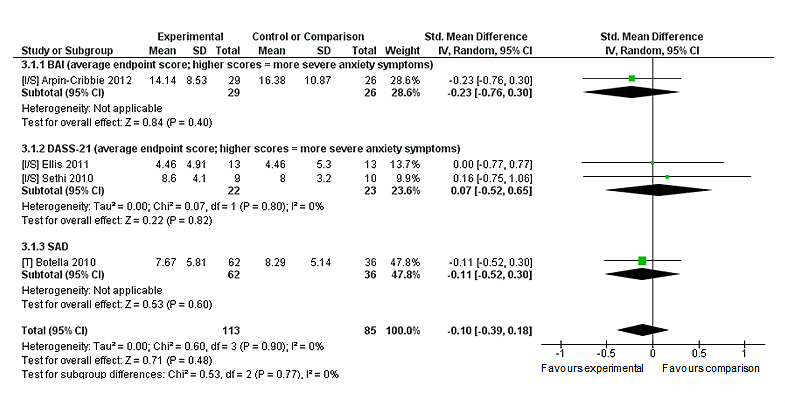
Sensitivity analysis of post-intervention anxiety outcomes for intervention compared to comparison intervention.

**Figure 8:**
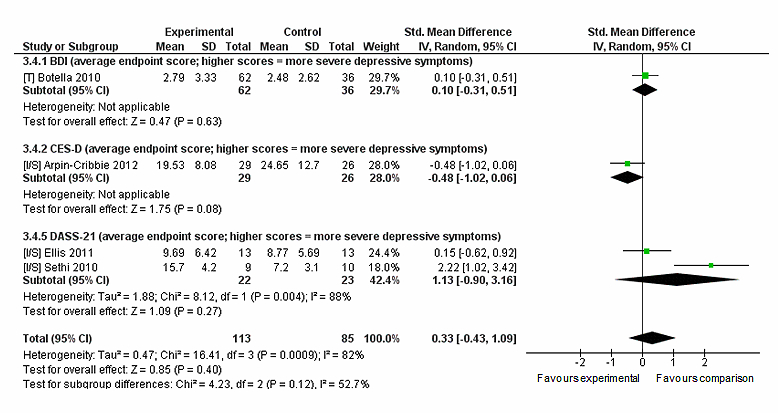
Sensitivity analysis of post-intervention depression outcomes for intervention compared to comparison intervention.

Five trials compared the intervention to a comparison intervention. Comparison interventions were a Web-based stress management intervention [28], a face-to-face version of the intervention [52], another computer-based CBT program [43], and an online support group [47]. Sethi’s trial [50] compared intervention to two comparison interventions consisting of face-to-face CBT and this combined with MoodGym. The face-to-face CBT was selected for this analysis to avoid double-counting of the intervention condition’s data. Outcome data from one trial could not be extracted for analysis [43], resulting in four trials, which all reported depression and anxiety outcomes, and included two trials of MoodGym [47,50]. Sensitivity analyses were conducted for both outcomes as only one trial in each outcome reported non-skewed data (see Multimedia Appendix 5). For anxiety, neither intervention nor comparison were favored over each other (n=198, 4 RCTs, pooled SMD −0.10, CI −0.39 to 0.18, *Z*=0.71, *P*=.48; *I* ^*2*^=0%, *P*=.90; see Figure 7). Likewise for depression outcomes neither condition was favored (n=198, 4 RCTs, pooled SMD 0.33, CI −0.43 to 1.09, *Z*=0.85, *P*=.40) (see Figure 8). There was a significant high level of heterogeneity reported for depression (*I* ^2^=82%, *P*=.001). Only one study reported outcomes relating to psychological distress (reported in Multimedia Appendix 5). There were no differences between conditions in leaving the study early (n=194, 4 RCTs, OR 1.18, CI 0.02-60.23, *Z*=0.08, *P*=.93; *I* ^2^=0%, *P*=.51). All attrition from the main intervention condition came from one study [52], wherein 32 participants left the study early. Seven (8.6%) in the comparison intervention condition left the study early.

### Additional Analyses

Given some of the methodological issues identified in the review, some additional sensitivity meta-analyses were performed. More recent publications appeared to report greater levels of methodological detail, possibly due to the research field being more established. The CONSORT-EHEALTH statement is a checklist providing a minimum list of recommendations for reporting RCTs of Internet and mobile-based interventions; it expands upon the previously published CONSORT statement [31,62]. The publication of the CONSORT-EHEALTH checklist was used as a benchmark for comparing ‘older’ (published ≤2011) to ‘newer’ (≥2012) publications. Included studies within the meta-analysis were separated based on their year of publication. These analyses could only be done for anxiety and depression outcomes in the intervention vs inactive control and vs comparison intervention comparisons due to low numbers of included trials and no differences in the publication dates in other comparisons and outcomes.

For depression outcomes in intervention compared to inactive control, a larger effect size was reported for more recent publications (n=164, 3 RCTs, pooled SMD −0.63, CI −0.94 to −0.31, *Z*=3.91, *P*<.001; *I* ^2^=0%, *P*=.70), than for older publications (n=548, 6 RCTs, pooled SMD −0.35, CI −0.60 to −0.09, *Z*=2.64, *P*=.008; *I* ^2^=47%, *P*=.09). For anxiety outcomes in the same comparison, there was little variation in the effect sizes and statistical significance in older (n=210, 4 RCTs, pooled SMD −0.60, CI −0.95 to −0.25, *Z*=3.37, *P*<.001; *I* ^2^=25%, *P*=.26) and newer publications (n=164, 3 RCTs, pooled SMD −0.55, CI −0.87 to −0.24, Z=3.46, *P*<.001; *I* ^2^=0%, *P*=.84). For depression and anxiety outcomes for intervention in contrast to a comparison intervention, there was only one post-2012 publication; analysis of all studies in this outcome is reported in the previous section. Looking at ≤2011 studies only, there was no difference reported between intervention and comparison for depression (n=143, 3 RCTs, pooled SMD 0.68, CI −0.33 to 1.69, *Z*=1.31, *P*=.19; *I* ^2^=82%, *P*=.004) or anxiety (n=143, 3 RCTs, pooled SMD −0.05, CI −0.39 to 0.28, *Z*=0.30, *P*=.76 (*I* ^2^=0%, *P*=.086).

Additional sensitivity analyses were also conducted to focus on trials that rewarded course credits for participation. This was not performed for the intervention vs active control comparison as all studies within this rewarded credit. Looking at studies that gave credit in the intervention vs inactive control comparison, the intervention was supported in improving anxiety outcomes (n=92, 3 RCTs, pooled SMD −0.75, CI −1.23 to −0.28, *Z*=3.10, *P*=.002; *I* ^2^=15%, *P*=.31) but not for depression (n=267, 4 RCTs, pooled SMD −0.16, CI −0.41 to 0.08, *Z*=1.33, *P*=.18; *I* ^2^=0%, *P*=.44). For studies that did not reward credit, intervention still supported anxiety (n=282, 4 RCTs, pooled SMD −0.51, CI −0.75 to −0.26, *Z*=4.07, *P*<.001; *I* ^2^=0%, *P*=.75) and also supported depression (n=282, 5 RCTs, pooled SMD −0.55, CI −0.78 to −0.32, *Z*=4.66, *P*<.001; *I* ^2^=26%, *P*=.25).

For participants who received credit in the intervention vs comparison intervention contrasts, neither condition was supported for anxiety (n=45, 2 RCTs, pooled SMD 0.07, CI −0.52 to 0.65, *Z*=0.22, *P*=.82; *I* ^2^=0%, *P*=.80) or depression (n=45, 2 RCTs, pooled SMD 1.13, CI −0.90 to 3.16, *Z*=1.09, *P*=.27; *I* ^2^=88%, *P*=.004). The same findings were repeated for participants that did not receive credit, upon anxiety (n=153, 2 RCTs, pooled SMD −0.15, CI −0.48 to 0.17, *Z*=0.93, *P*=.35; *I* ^2^=0%, *P*=.73) and depression outcomes (n=153, 2 RCTs, pooled SMD −0.16, CI −0.73 to 0.40, *Z*=0.57, *P*=.57; *I* ^2^=65%, *P*=.09).

## Discussion

### Principal Findings

A total of 17 studies were retrieved for this review, of which 14 were entered into meta-analysis. The majority of studies administered measures of both depression and anxiety (9/17, 53%), with two also measuring stress or psychological distress. Two studies reported targeting depression alone, with the six remaining studies reporting a mixture of outcomes. The majority were Web-based trials (n=13) with four delivered via an offline computer-delivered program. The review findings suggest Web-based and computer-delivered interventions can produce beneficial mental health outcomes in university students, supporting previous reviews of Internet and computerized interventions for depression and anxiety [14,16,40]. Our search found several recent publications not reviewed previously [4], which demonstrates the fast pace of publications in this field.

Findings demonstrated a difference in outcome data depending on the type of analyses conducted. Non-skewed data alone did not favor intervention in improving anxiety, but sensitivity analysis favored intervention when compared to inactive control. However, improvements in anxiety outcomes were not supported when intervention was compared to active control or comparison intervention. Similar findings were reported for depression outcomes. Non-skewed data for intervention compared to inactive control revealed a larger effect size (SMD −0.67) than the sensitivity analysis (SMD −0.43), suggesting skewed data can potentially affect the overall power of interventions. For psychological distress, the data did not support the intervention. The small number of studies, the different measures used, and the type of intervention complicates interpretation of findings. For stress, compared to inactive control, both meta-analyses supported intervention, with a larger effect found for sensitivity (SMD −0.73) than non-skewed analysis (−0.44). Similarly, the heterogeneity went from 0% for non-skewed analysis to 70% for sensitivity analysis, so this difference could be due to the skewed data.

When compared to inactive control, interventions appeared to be supported in improving outcomes apart from psychological distress. When compared to active control and comparison interventions, computer-delivered and Web-based interventions were not significantly supported in improving depression or anxiety. This was anticipated given that participants were still actively doing something, compared to an inactive control [40]. Neither intervention nor comparison intervention were significantly favored in meta-analysis, which may suggest some equivalency in their effect upon improving anxiety and depression outcomes. A reason this finding may have occurred could be due to the type of comparison intervention used. Two comparison interventions were face-to-face CBT, which is representative of the kind of help university students would typically receive for common mental health problems. Further research comparing these technology-based interventions to treatment-as-usual conditions would be beneficial in exploring the viability of self-guided Internet-based interventions for university students, and whether they have equivalency in comparison to the therapies young people would usually receive. Larger effect sizes within intervention vs inactive control comparisons than intervention vs active control have been reported previously in CCBT reviews [16,40]. Both active controls were identical in their content; the lack of significant effect found in the meta-analysis suggests neither intervention nor active control were more advantageous in improving outcomes. This finding may question what is the minimum level of active control needed to produce positive change.

Moderate to high heterogeneity were reported for two of the analyses comparing intervention against active control and comparison intervention. This could be due to the type of comparison intervention or that differences in outcome data at baseline affected post-intervention symptom improvement. Grist and Cavanagh [16] identified type of control condition as being a significant moderating factor explaining heterogeneity within meta-analyses. In trials of CCBT, active controls often share some commonalities with the experimental intervention; effect sizes reported previously suggest CCBT can offer some additional small benefits in improving psychological outcomes [16]. A total of 13 studies involved CBT-based interventions, which supports findings from previous CCBT reviews [14-17]. While this continues to provide strong support for CCBT, research should explore what other evidence-based psychological and psychotherapeutic theories can be adapted to this medium [40]. It is difficult to determine which elements of the intervention produced the most beneficial effects, and there are many factors to consider, such as level of support, intervention length, the number and content of modules, and actual participant engagement.

Separating older and newer studies did appear to have an effect upon the effect sizes for depression outcomes in intervention vs inactive control comparisons, with a larger effect size found for more recent publications. Within the same comparison, there was little difference in effect sizes for anxiety, and separating the studies did not appear to add any additional insight into intervention vs comparison intervention analyses. These contrasting findings may suggest research into Internet interventions has somewhat strengthened over the years and become more methodologically sound. However, these links are tenuous given the small numbers of included trials within the separate analyses.

Future trials within university student populations should consider the effect of participant incentives and rewards upon outcomes; given that students are typically financially strained, outcomes in trials may differ from their real-world non-trial use of interventions. Separate sensitivity analyses were conducted to explore whether receiving participatory reward affected outcomes. Within the intervention vs inactive control comparison for anxiety, a larger effect size was reported for studies that did reward credit (SMD −0.75) than for those that did not (SMD −0.51). However, for depression the analysis did not support intervention in studies that rewarded credit, whereas those that did not use incentives reported a significant favoring for intervention (SMD −0.55). Sensitivity analyses for rewards within the intervention vs comparison intervention contrast reported similar findings in line with the main meta-analysis. The contrasting findings for this comparison do not allow us to precisely conclude that rewarding participants does increase an intervention’s efficacy, but incentives and rewards are a factor to consider when disseminating trial findings. A meta-analysis of Web-based surveys found that incentives for participation increased individuals’ motivation to start and complete the survey [63]. Similarly, college students who participated in an incentive-based online intervention for weight loss reported that financial rewards acted as a strong external motivator to lose weight and achieve weekly goals, although they also commented that the financial incentive did not influence their intrinsic motivation to participate [64]. The majority of studies that utilized participatory reward did so through providing course credit. This may differ somewhat from financial incentives but nonetheless requires consideration as students may place similar personal value upon monetary and course credit rewards. Some publications insufficiently reported their outcome data. Authors should aim to provide a CONSORT-EHEALTH statement to help report their interventions [31] so the design and content of interventions can be viewed clearly. Authors in more recent publications appeared to report more aspects of this checklist in their respective publications.

Participant dropouts were reported in 12 studies; attrition is common in these types of intervention trials [65,66]. Two studies [50,54] had similar rationales for delivering their interventions over a short timeframe—shorter interventions are associated with increased engagement and retention of participants. Baseline symptoms have been associated with attrition rates—lower depressive symptoms were positively associated with increased adherence to interventions in one review [65]. As some of the included interventions recruited participants with minimal/mild symptomology, this is an issue to consider. Only two trials assessed whether participants’ levels of adherence affected their level of post-intervention improvement upon mental health, in which no associations were found [48,53]. With Internet-delivered interventions, it can be difficult to assess participants’ levels of intervention engagement and there may be variation in how participant engagement is defined [31].

Participant attrition was more likely to occur in intervention groups when compared to inactive control, with no association found for comparisons to active control or comparison intervention. This was found in a review of CCBT [16]. Grist suggests the finding of no attrition differences in intervention and active control groups indicates that attrition is common in any active condition, whether it be the experimental intervention or an active control, and is not just a consequence of receiving CCBT. It may suggest some level of support is required to help participants adhere to the intervention. Only a few trials provided detail about participants’ reasons for dropping out. Attrition has commonly been used as a proxy measure of participant evaluation and attitudes towards CCBT [20,48]. Interventions that do not sufficiently engage or appeal to the user may be more susceptible to dropout [48]. Interventions could potentially show positive effects due to the unengaged participants withdrawing from the study [57]; attrition may partially account for this review’s positive findings. Seeking participants’ reasons for disengaging from intervention is important in helping identify factors affecting adherence.

Aside from Botella’s trial, which aimed to treat diagnosable social phobia [52], none of the studies explored post-intervention diagnosis of mental disorders. This is important as these interventions are used as mental health prevention and longitudinal follow-up would allow us to explore the interventions’ preventative effects. Help-seeking intentions and/or behaviors were not assessed through standardized measures in any study; these interventions can subsequently affect participants’ help-seeking [40]. Over a third of participants in one trial stated that as a result of the intervention they had changed their behavior, which included seeking out more information, trying self-help techniques described in the intervention, and supporting others [49]. It is understandable that follow-up may be difficult in university students given the transient nature of university life—students may change address or leave higher education between post-intervention and follow-up periods. The timing of conducting trials is important given the fluctuating demands occurring during the academic year. Only three studies reported when post-intervention measures were administered; two of these were during examination periods [44,49] and so improvements may also be demonstrated during periods of high stress.

Just over half the interventions were semi-guided. Most of these incorporated a strategy to maintain engagement and thereby encourage adherence, such as using standardized reminders, receiving the intervention at a study site, or support from a non-therapeutic individual. We did not analyze whether there were any differences in effects between semi-guided and self-administered interventions, and cannot make assumptions about the impact of human interaction upon intervention effectiveness. A previous review found larger effect sizes for self/un-guided interventions than ones involving guidance [16].

Two interventions [46,55] had a large amount of human contact with participants. In both trials, participants received weekly contact from researchers or from program coaches to support them in completing the intervention. This kind of support provides reduced training costs compared to interventions that involve support from health care professionals, and as the program coaches were students themselves, participants may have found them relatable. Administration of trials in researcher-monitored settings could have affected participants’ engagement with the intervention [14]. Johannson and Andersson [34] found increased human therapeutic support given to users was significantly associated with larger intervention effects. There was limited evaluation regarding participants’ perceptions about the beneficial or therapeutic effects of human support, but nonetheless the amount of contact participants had with another person could affect intervention effectiveness.

Mental health outcomes were assessed using a small number of well-established continuous measures aligned with diagnostic criteria. This made comparisons in the meta-analysis less complicated; however, having several measures can increase statistical heterogeneity [67]. We attempted to counteract this by investigating intervention effects by subgrouping each type of measure within each outcome, and looking separately at the overall pooled effect. By doing this, we could explore measurement comparisons for each outcome, which did show some variation in the different measures used for the same outcomes.

The overwhelming presence of skewed data in the included studies affected the quality of the available evidence. Skewed data has been reported previously in a review of computer-delivered interventions for reducing alcohol consumption [68]. Almost all included studies reported the mean and standard deviation from outcome measures, and none reported alternative measures of central tendency. Only a minority had transformed skewed data or used non-parametric tests. The meta-analyses reported a vast quantity of heterogeneity, which hinders their generalizability, and the differences in the scoring range of measures may be a reason why it occurred. For example, the two psychological distress measures varied on their scoring range: the PHQ-4 (Patient Health Questionnaire) was a brief measure where scores range from 0 to 12, while scores on the K10 (Kessler Distress Scale) range from 0 to 40. Large heterogeneity has been reported previously in reviews of Internet-delivered and computer-based interventions for depression [40,69]. Richards and Richardson [69] suggest eligibility criteria can be a cause of heterogeneity. This is possible given the variation in the baseline symptomology eligibility criteria of included participants. Some trials recruited participants experiencing minimal to moderate levels of depression, anxiety, or stress [45-47,50]; within some of the same analyses, there were participants who were included if they were experiencing elevated symptoms [48,49]. This variation in symptomology may affect the overall power of the included interventions.

Small sample sizes were apparent. The smallest sample involved 38 participants, within which there were four arms, of which two contained nine participants each [50]. There was limited detail about power calculations to recruit appropriate sample sizes. The forest plots show studies with smaller samples were associated with larger confidence intervals and are less reliable than larger samples. Coupling this with the considerable skew means the findings need to be approached with caution. Completers analysis may bias the calculated effectiveness of interventions as these analyses are likely to produce larger outcome effects [70]. ITT analysis helps avoid selection bias that can occur if only those completing measures at all study time-points are analyzed [71].

The use of participation reminders requires consideration. Interventions trialed in the included studies may not have reminders when administered in a non-trial context. Three studies trialed MoodGym, a freely available online resource that any member of the public can sign up to. In this context, general public users do not receive reminders to complete the intervention—unlike in two included studies [47,50] where participants completed it in a monitored setting.

Funnel plots were briefly inspected to explore possible presence of publication bias; these did not appear to show any unusual asymmetry. This was approached with caution as funnel plot asymmetry should ideally be used when ≥10 studies are in analysis [72]. The majority of studies reported positive outcomes on at least one relevant mental symptomology measure. We did not include non-peer reviewed studies and so did not include unpublished data. As reported previously by Farrer [4], not all may have been designed for university students—instead they were sampled to opportunistically trial out the intervention and they may have some differences to the ideal target population. Participants in some studies were already experiencing minimal symptoms upon enrolment, meaning it is problematic to determine how much of an effect the intervention had upon reducing developmental risk of ill mental health. For example, intervention participants in one trial [44] reported a mean pre-post intervention decline of <3 points on the BDI (Beck Depression Inventory); at baseline, participants were already classified as having minimal depressive symptoms. It is difficult to address the significance of this small decrease in already minimal symptomology, and the preventative effect of interventions is further complicated by limited follow-up. No studies assessed utilization of mental health services or diagnosis of mental disorders as an outcome measure, making it difficult to know if interventions reduced the risk of developing a mental disorder or affected mental health service use. For the meta-analyses, only post-intervention short-term data were used due to limited long-term follow-up. We are unsure about the long-term maintenance of improvements in outcomes.

Participants in seven studies received course or financial credit for participation [42-45,47,50,51,55] and eight samples were recruited from psychology degree courses. In sensitivity analyses, one comparison for depression (interventions vs inactive control) did not support the intervention, whereas it did in the overall analysis. This may bias findings as those who participated for credit are likely be different from students who seek help without an reward incentive for doing so. Likewise psychology students may be more knowledgeable about mental health and the trial process, and thus more receptive to interventions. However, the effects may be greater in students who were not aware of the possibilities of CBT/evidence-based approaches to improve mood. The overrepresentation of psychology students may account for the gender imbalance in recruitment [73]. Young male adults are frequently cited as being less likely to seek out help for their mental health [74,75], and it has been suggested Internet-based interventions could reach out to men [75]. Researchers need to reach out to students in other disciplines and also recruit more males to their trials. Another factor to consider relates to the age range of participants. Unlike Farrer [4], we did not have age as inclusion criteria for the review. The average age calculated from 15 included studies was 22.6 years, and some samples included older adults. This deviates from the traditional age range of university students, and older students may have different mental health needs than typically aged students. Given this, the findings may not be fully generalizable to younger students. Future research would benefit by focusing on sampling students within the 18-25 year age range typical of student populations, or consider age as a moderating factor of intervention effectiveness within this population.

A moderate risk of bias was calculated mostly due to insufficient details reported about trial methodology and outcome measures, meaning we were unclear about several risk of bias outcomes. Only a minority of studies reported their randomization method; this has been reported previously in reviews of CCBT, technology-based interventions, and interventions to improve help-seeking and stigmatizing attitudes and beliefs in university students [4,16,23,76]. Grading the blindness of participants in included studies may be irrelevant given the nature of the types of intervention and trial design [40]. Some studies insufficiently reported their data, which affects the quality of the available evidence. Reporting methodological factors, such as randomization method, concealment, and the blinding of research personnel, is essential to judging trial quality. Researchers in this field are becoming more aware of using CONSORT-EHEALTH guidelines in their publications [31], which addresses several of these methodological factors.

While all included studies explored the statistical significance of outcome data, only a few looked into whether improvements were clinically significant. The few that calculated these found intervention participants showed a higher level of reliable and clinically significant improvement compared to controls [28,46,52,57]. Calculating this provides additional value about the recovery status of participants. It would also be useful to explore whether the improvements reported in the outcome measures correspond to participants’ perceptions, as there has been disagreement between severity of symptoms reported on a common depression measure and participants’ actual verbal description of symptom severity [77]. This could be done by asking them whether they felt the intervention helped their mental well-being, and might help to address the apparent overreliance on focusing on psychometric measures. One qualitative study found students felt use of an online resource helped them manage their mental well-being during periods of psychological distress [78].

### Implications for Practice

As the intervention vs comparison intervention analyses suggested some level of equivalence in outcomes, individuals working in student health, such as welfare advisors and counsellors, may be considering online and technology-based resources they can use to support their students. Some universities do appear interested in using online resources, as several British HEIs have incorporated Web-based interventions into their welfare services, such as the “CALM/Relief” series [79]. None of the included studies assessed whether these interventions had outcomes upon students’ academic performance. This is likely to be an important outcome for policymakers given the reputation of their institutions. The best improvements in mental health outcomes may be achieved by combining self-help with face-to-face support [19].To help address the increased demand for university-based counselling, online resources could be used as a support tool by university students while waiting to see a relevant professional [78]. Similarly, these resources could also be used as an adjunct by students in between counselling appointments.

### Implications for Research

Future research needs to consider sufficient sample sizes required for trials, and address the skewed data present in outcome data by either transforming it or using alternative tests. Measurements of help-seeking intentions and behavior, as well as aspects of mental health literacy, would be highly useful in future research as online interventions are often promoted as an alternative to seeking face-to-face help or preventing onset of ill mental health [23]. Researchers would benefit from collaborating with the student population to understand what measurable outcomes are important to them; as these young people are in higher education to obtain a qualification, it is expected that academic performance and retention would be salient outcomes. Mental health difficulties can significantly impair students’ academic performance and social functioning; future research should incorporate outcomes reflecting these domains. Gaining user evaluation of interventions through qualitative methods such as interviews and focus groups would also be highly useful in attaining feedback to address the worth of the intervention and to make interventions more appropriate for student needs [20].

### Limitations

All studies were coded by one author (EBD) and were discussed as necessary with CG. The use of one coder may have unintentionally biased the results. There is the possibility that relevant publications may have been missed in the search. However, the search was conducted on several databases and updated through a repeat search, as it had taken some time to conduct the review. Likewise, Farrer’s review [4] was searched for additional publications. For meta-analysis, we could not extract data from three included trials, meaning the pool of data from included interventions was smaller. Similarly for the anxiety meta-analyses, measures that may reflect certain distinct aspects of anxiety disorders, such as anxiety sensitivity and social anxiety, were incorporated into one analysis for all anxiety outcomes, which may also have induced bias. The studies trialing the same three interventions had slight variation in how they individually conducted and how participants accessed the intervention. Lintvedt [49] coupled MoodGym with an information-only website, meaning participants received additional information not delivered in the other MoodGym trials [47,50]. The type of intervention may have influenced the reported heterogeneity. In their meta-analysis of Internet-delivered CBT for depression and anxiety, Spek [14] found higher heterogeneity in treatment interventions compared to ones focused on prevention. For our review, there was only one intervention that could clearly be defined as treatment; however, there was variation in the type of universal and selective/indicated interventions being trialed. The level of human support and contact within included interventions is another aspect affecting participant-intervention engagement, which may have impacted effect sizes [14].

Trials of mobile apps for improving mental health outcomes were not included in this review, as it was felt these were still an emerging technology at the time. University students may be a group likely to use apps as they also present many of the same benefits as computer-based/Web-based interventions, but could be more accessible given the popularity of smartphones and tablets. Farrer’s review [4] was explored for app-based interventions. A recent review of mental health apps for smartphones/tablets found only five apps that had been trialed [80], one of which was trialed on a student population [81]. However, as found with several in the present review, this trial’s methodology and data were not reported clearly and it is unclear whether the intervention was a smartphone app.

Several studies analyzed conducted completers analyses, which may bias review findings as these analyses are likely to produce larger outcome effects [70]. All interventions used different content and multimedia, which could affect how much participants interacted with the intervention and subsequently their effectiveness [23]. It is difficult to know whether improvements produced by both intervention and active control conditions would have been maintained in the long-term due to limited follow-up. Given that some active controls/comparison interventions produced similar outcome effects to the intervention being trialed, consideration is needed regarding the minimum intervention needed to produce effective change in outcomes. Use of active controls may result in difficulty in understanding the true effect of the experimental intervention upon outcomes [70].

Interventions from different theoretical approaches were combined together for the meta-analysis. Limited numbers of non-CBT trials meant separate analyses exploring different approaches could not be conducted. Although there were only a small number of non-CBT trials within meta-analyses, this could potentially skew findings and so future reviews may want to separately analyze outcomes based on the theoretical underpinning of interventions. Random Effects Models were used for all analyses; however, this may induce bias as it places larger significance on smaller studies [82]. Many trials involved small samples, meaning this bias may have occurred. Finally, no-treatment control and wait-list controls were collapsed into one comparison category (inactive control) for analysis. There were seven trials using wait-list and four using a no-treatment control. This could affect findings as those assigned to wait-list control would have been expecting to receive intervention at some point and may show improvements in their symptomology due to expectation effects.

### Conclusions

Overall, this review provides some cautious findings that suggest online and computer-delivered interventions can potentially be beneficial in improving depression, anxiety, and psychological distress outcomes in university students. These interventions are not a panacea for all, but do provide an easily implemented health promotion and prevention strategy that can be easily reached by university students. The benefits of these interventions may potentially help HEIs in promoting good mental health and well-being to its population and support students’ academic performance [83]. However, trials in this review did not assess students’ academic performance before or after receiving intervention. The findings support the effectiveness of the adaptation of CBT into self-guided, Internet-delivered interventions. However, several methodological shortcomings, including small sample sizes and a large amount of skewed data, mean the findings need to be treated with a high degree of caution. As concluded in a meta-analysis of psycho-educational mental health interventions [70], there needs to be more investigation into the factors influencing intervention effectiveness. Further participant feedback is encouraged to evaluate online and computer-based interventions and to help further tailor interventions to university student populations.

## Multimedia Appendix 1

Search terms used in online databases.

## Multimedia Appendix 2

List of studies excluded from the review.

## Multimedia Appendix 3

PRISMA checklist.

## Multimedia Appendix 4

Summary of Web-based and computer-delivered interventions to improve depression, anxiety, psychological distress, and stress conducted in university student populations included in the present review.

## Multimedia Appendix 5

Non-skewed data that could not be incorporated into meta-analyses due to being sole study for specific outcomes of interest.

## Abbreviations

ASI: Anxiety Sensitivity Inventory
BAI: Beck Anxiety Inventory
BDI: Beck Depression Inventory
CBT: Cognitive Behavioral Therapy
CCBT: Computerized Cognitive Behavioral Therapy
CES-D: Center for Epidemiologic Studies Depression Scale
CI: confidence interval
DASS-21: Depression Anxiety and Stress Scale – 21 item version
HEI: higher education institution
ITT: intention-to-treat
K-10: Kessler Distress Scale – 10 item version
PHQ-4: Patient Health Questionnaire – 4 item version
PRISMA: Preferred Reporting Items for Systematic Reviews and Meta-Analyses
PSS: Perceived Stress Scale
RAM: Random Effects Model
RCT: Randomized Controlled Trial
SAD: Social Avoidance and Distress scale
SMD: Standardized Mean Difference
TAI: Test Anxiety Inventory
[U]: universal intervention
[I/S]: indicated or selective intervention
[T]: treatment intervention
