# Supplementary material for: Computer-Delivered and Web-Based Interventions to Improve Depression, Anxiety, and Psychological Well-Being of University Students: A Systematic Review and Meta-Analysis
Source: J Med Internet Res. 2014 May 16;16(5):e130. doi: 10.2196/jmir.3142 (PMC4051748; doi:10.2196/jmir.3142)
Supplement: Supplementary file 1 [file jmir_v16i5e130_app1.pdf]

Multimedia Appendix 1. Search terms used in online databases (except publisher websites and Google Scholar). Four separate search strings were performed in each database.

Search string	Type of intervention		Target change of intervention		Sample
Search 1	"online intervention" *OR* "internet intervention" *OR* "internet based intervention" *OR* "web intervention" *OR* "web based intervention" *OR* "online treatment" *OR* "computer based interventions" *OR* "computer intervention"	*AND*	"behaviour change" *OR* "behavior change" *OR* "behaviour modification" *OR* "behavior modification" *OR* "health behaviour" *OR* "health behavior"	*AND*	"undergraduate student" *OR* "college student" *OR* "university student" *OR* "student" *OR* "higher education" *OR* "tertiary education"
Search 2	"intervention" *AND* ("online" *OR* "web" *OR* "computer" *OR* "internet")	*AND*	"behaviour change" *OR* "behavior change" *OR* "behaviour modification" *OR* "behavior modification" *OR* "health behaviour" *OR* "health behavior"	*AND*	"undergraduate student" *OR* "college student" *OR* "university student" *OR* "student" *OR* "higher education" *OR* "tertiary education"
Search 3	"online intervention" *OR* "internet intervention" *OR* "internet based intervention" *OR* "web intervention" *OR* "web based intervention" *OR* "online treatment" *OR* "computer based interventions" *OR* "computer intervention"	*AND*	"sexual health" *OR* "condom" *OR* "HIV" *OR* "alcohol" *OR* "drug" *OR* "substance use" *OR* "cannabis" *OR* "smoking" *OR* "smoking cessation" *OR* "physical activity" *OR* "exercise" OR "obesity" *OR* "weight" *OR* "eating"	*AND*	"undergraduate student" *OR* "college student" *OR* "university student" *OR* "student" *OR* "higher education" *OR* "tertiary education"
Search 4	"online intervention" *OR* "internet intervention" *OR* "internet based intervention" *OR* "web intervention" *OR* "web based intervention" *OR* "online treatment" *OR* "computer based interventions" *OR* "computer intervention"	*AND*	"mental health" *OR* "depression" *OR* "depressive symptoms" *OR* "anxiety" *OR* "affective disorder" *OR* "bipolar" *OR* "psychosis" *OR* "social anxiety" *OR* "exam anxiety" *OR* "stress" *OR* "perfectionism" *OR* "personality" *OR* "insomnia" *OR* "sleep" *OR* "eating disorders" *OR*	*AND*	"undergraduate student" *OR* "college student" *OR* "university student" *OR* "student" *OR* "higher education" *OR* "tertiary education"

	"anorexia" *OR* "bulimia" *OR* "eating problems" *OR* "addiction" *OR* "gambling" *OR* "self harm" *OR* “self-harm” *OR* "suicide" *OR* "mental health difficulties"	

Databases searched:

Science Direct

CINAHL

ASSIA

SCOPUS

Cochrane Central Library of Controlled Trials (CENTRAL)

EMBASE

PubMed

Web of Science

MedLine

Google Scholar (reduced search terms used)
