# Supplementary material for: Computer-Delivered and Web-Based Interventions to Improve Depression, Anxiety, and Psychological Well-Being of University Students: A Systematic Review and Meta-Analysis
Source: J Med Internet Res. 2014 May 16;16(5):e130. doi: 10.2196/jmir.3142 (PMC4051748; doi:10.2196/jmir.3142)
Supplement: Supplementary file 2 [file jmir_v16i5e130_app2.pdf]

Multimedia Appendix 2. List of studies excluded from the review.

Published studies that were identified in the search process and were related to mental health. Upon reading the full-texts, these were subsequently excluded and are presented below alongside the reasoning for their exclusion.

**Publication**	**Reason for exclusion**
Cukrowicz KC, Smith PN, Hohmeister HC, Joiner TE: **The moderation of an early intervention program for anxiety and depression by specific psychological symptoms.** Journal of Clinical Psychology 2009, 65(4):337-351.	Same sample as Cukrowicz & Joiner (2007), article focused on moderating factors in experimental condition.
Currie SL, McGrath PJ, Day V: **Development and usability of an online CBT program for symptoms of moderate depression, anxiety, and stress in post-secondary students.** Computers in Human Behavior 2010, 26(6):1419-1426.	Feasibility study prior to trial; not RCT
Davis-McCabe C, Winthrop A: **Computerised CBT: University students experiences of using an online self-help programme.** Counselling Psychology Review 2010, 25(4):46-55.	Qualitative evaluative study; no RCT conducted/reported in article.
Finkelstein J, Lapshin O: **Reducing depression stigma using a web-based program.** International Journal of Medical Informatics 2007, 76(10):726-734	Not administered to university students, not an RCT
Freeman E, Barker C, Pistrang N: **Outcome of an online mutual support group for college students with psychological problems.** CyberPsychology & Behavior 2008, 11(5).	Was RCT but only two conditions - same intervention trialled in both with no no-intervention condition.
Givi HG, Imani H, Agh A, Rik NM, Mehrabadi S: **Efficiency of computerized cognitive behavioral therapy versus clinical intervention for the treatment of major depression.** [Farsi] Koomesh 2012, 13(2):218-224.	Article translated from Farsi to English, however there were difficulties understanding translated article. Author was contacted but no response.
Haas A, Koestner B, Rosenberg J, Moore D, Garlow SJ, Sedway J, Nicholas L, Hendin H, Mann J, Nemeroff CB: **An interactive web-based method of outreach to college students at risk for suicide.** Journal of American College Health 2008, 57(1):15-22.	Intervention did not meet intervention criteria for review: intervention was an online screening tool for counselling service use.

Horgan A, McCarthy G, Sweeney J: **An Evaluation of an Online Peer Support Forum for University Students With Depressive Symptoms**. Archives of Psychiatric Nursing 2013, 27(2):84-89.	Intervention not trialled as an RCT; only one trial condition.
Lange A, Ven J-PQRvd, Schrieken BAL, Bredeweg B, Emmelkamp PMG: **Internet-mediated, protocol-driven treatment of psychological dysfunction.** Journal of Telemedicine and Telecare 2000, 6(1):15-21.	Intervention did not meet intervention criteria for review: intervention was platform for therapist-involved (guided) therapy
Lau KS, Siong KH, Tang HY, Cheng PW, Cheung KS, Chan SW, Lee PWH, Wong JGWS: **An innovative web-based peer support system for medical students in Hong Kong.** Medical Teacher 2007, 29(9-10):984-986.	Not RCT, no comparison group, no mental symptomology measures.
Littleton H, Buck K, Rosman L, Grills-Taquechel A: **From Survivor to Thriver: A Pilot Study of an Online Program for Rape Victims.** Cognitive and Behavioral Practice 2012, 19(2):315-327.	Not an RCT and no comparison group used.
Mailey EL, Wójcicki TR, Motl RW, Hu L, Strauser DR, Collins KD, McAuley E: **Internet-delivered physical activity intervention for college students with mental health disorders: a randomized pilot trial.** Psychology, Health & Medicine 2010, 15(6):646-659.	Intervention did not meet intervention criteria for review: intervention involved face-to-face meetings and tailored emails between participant and counsellor.
Mitchell N, Dunn K: **Pragmatic evaluation of the viability of CCBT self-help for depression in higher education.** Counselling & Psychotherapy Research 2007, 7(3):144-150.	Not an RCT and no comparison group used.
Rassau A, Arco L: **Effects of chat-based on-line cognitive behavior therapy on study related behavior and anxiety.** Behavioural and Cognitive Psychotherapy 2003, 31(3):377-381.	Case study detailing one student
Richards D: **Features and benefits of online counselling: Trinity College online mental health community.** British Journal of Guidance & Counselling 2009, 37(3):231-242.	Described intervention development only. No RCT conducted.
Richards D, Tangney B: **An informal online learning community for student mental health at university: A preliminary investigation.** British Journal of Guidance &	Not an RCT and no outcome data used

Counselling 2008, 36(1):81-97.	
Su W, Fang X, Miller JK, Wang Y: **Internet-based intervention for the treatment of online addiction for college students in China: A pilot study of the Healthy Online Self-Helping Center.** Cyberpsychology, Behavior, and Social Networking 2011, 14(9):497-503.	Was RCT but intervention did not meet intervention criteria for review: intervention focused on internet addition, the administered measure assessed internet dependency behaviour and not mental state.
Tillfors M, Carlbring P, Furmark T, Lewenhaupt S, Spak M, Eriksson A, Westling BE, Andersson G: **Treating university students with social phobia and public speaking fears: internet delivered self-help with or without live group exposure sessions.** Depression and Anxiety 2008, 25(8):708-717.	Was RCT but only two conditions - same intervention trialled in both with no no-intervention condition.
Trockel M, Manber R, Chang V, Thurston A, Taylor CB: **An e-mail delivered CBT for sleep-health program for college students: effects on sleep quality and depression symptoms.** J Clin Sleep Med 2011, 7(3):276-281.	Not an RCT: participants were not randomised to the two conditions - assignment based on current residence.
