# Supplementary material for: Computer-Delivered and Web-Based Interventions to Improve Depression, Anxiety, and Psychological Well-Being of University Students: A Systematic Review and Meta-Analysis
Source: J Med Internet Res. 2014 May 16;16(5):e130. doi: 10.2196/jmir.3142 (PMC4051748; doi:10.2196/jmir.3142)
Supplement: Supplementary file 4 [file jmir_v16i5e130_app4.pdf]

Multimedia Appendix 3. Summary of web-based and computer-delivered interventions to improve depression, anxiety, psychological distress and stress conducted in higher education populations included in the present review.

Study	***Design, arms (N per arm), N, location***	***Sample demographics and inclusion criteria***	***Intervention***	***Length/dosage***	***Location***	***Outcome measures***	***Analysis***
Arpin-Cribbie et al 2012	RCT<br>3 arms: Intervention (29), CI (26), WLC (25)<br>N=77<br>One HEI, Canada	Selective/indicated<br><br>Undergraduate psychology students<br>70% female, M age 20.1yrs<br>High levels of perfectionism (PCI)	Internet-delivered CBT for perfectionism<br><br>Self-administered	13 modules over 10 weeks; suggested order for working through intervention (not mandatory)	Own location	CES-D<br>ATQ<br>BAI<br>ASI<br>PSS<br>MPS-F – CM<br>MPS-HF<br>PCI	Completers
Botella et al 2010	RCT<br>3 arms:<br>Intervention (62), CI (36), WLC (29)<br>N=127<br>Two HEIs, Spain	Treatment<br><br>Undergraduate and postgraduate students<br>61 females, M age 24.4yrs (completers only)<br>Met DSM-IV diagnostic criteria for social anxiety/phobia	*"Talk to Me"*: website-based CBT for fear of public speaking<br><br>Self-administered	Available over 8 weeks, participants exposed to anxious situation through use of embedded videos	Own location	SAD<br>BDI<br>BFNE<br>FPSQ<br>SSPS<br>MS	ITT
Braithwaite & Fincham 2007*	RCT<br>3 arms: Intervention, CI and AC<br>N=91<br>One HEI, USA	Universal<br><br>Undergraduate psychology students<br>59% female<br>In romantic relationship of ≥4 months duration	*ePREP*: relationship functioning intervention<br>Based on CBT<br>Delivered via computer<br><br>Semi-guided	1-hour computer session followed by weekly standardised emails over 8 weeks☐<br>Participants had paper copy of computer intervention	Researcher-monitored laboratory	BAI<br>BDI<br>PANAS<br>CTS-2<br>PRQC<br>CPQ-CC<br>Trust scale	Uncertain
Braithwaite & Fincham 2009	RCT<br>2 arms: Intervention (38), AC (39)<br>N=77<br>One HEI, USA	Universal<br><br>Undergraduate psychology students<br>71% female, M age 19.4yrs<br>In romantic relationship of ≥4 months duration	*ePREP*: relationship functioning intervention (same as Braithwaite & Fincham 2007)<br><br>Semi-guided	Same as Braithwaite & Fincham (2007)	Researcher-monitored laboratory	BAI<br>BDI<br>CTS-2<br>CSI	ITT

Cavanagh et al 2013	RCT<br>2 arms:<br>Intervention (54), WLC (50)<br>N=104<br>One HEI, UK	Universal<br>Undergraduate and postgraduate students<br>92 females, M age 24.7yrs	"*Learning Mindfulness Online*": intranet-based website designed to teach Mindfulness<br><br>Semi-guided	Available over 2 weeks, 4 standardised reminder emails sent over period	Own location	PSS<br>PHQ-4<br>FFMQ	ITT
Chiauzzi et al 2008*	RCT<br>3 arms: Intervention (80), AC (80), NTC (80)<br>N=77<br>Six HEIs, USA	Selective/indicated<br><br>Undergraduate students<br>123 females<br>18-24yrs, experiencing elevated perceived stress (PSS)	*My StudentBody-Stress*: psycho-educational student-tailored website for stress management - possibly based on cognitive therapy<br><br>Self-administered	≥20 minutes at least four times over two week period	Own location	PSS<br>HPLP-II<br>CAS	Uncertain
Cukrowicz & Joiner 2007	RCT<br>2 arms: Intervention (81), AC (71)<br>N=238; N=152 analysed<br>One HEI, USA	Selective/indicated<br><br>Undergraduate psychology students<br>169 female, M age 19.2yrs<br>Minimal/mild symptoms of depression and anxiety (BDI, BAI)	*CBASP*: psycho-educational and CBT for depression and anxiety<br><br>Delivered via computer<br><br>Semi-guided	2-hour computer session followed by weekly standardised emails over 8 weeks<br><br>Eight worksheets based on Situational Analysis - participants to work through these when encounter stressful situation.	Researcher-monitored laboratory	BAI<br>BDI<br>PANAS<br>STAI-S	Completers
Day et al 2013	RCT<br>2 arms:<br>Intervention (33), WLC (33)<br>N=66; N=66 analysed<br>Three HEIs, Canada	Selective/indicated<br><br>Undergraduate and postgraduate students<br>59 females, M age 23.55yrs<br>Mild/moderate symptoms of depression or anxiety or stress (DASS-21)	"*Feeling Better*", website-based CBT program<br><br>Semi-guided	5 core modules, plus 6 optional modules.<br>Completed in own time over 6 week period.<br><br>Participants received weekly email or telephone non-therapeutic support from a program coach - this was to monitor and support completing intervention.	Own location	DASS-21	ITT

Ellis et al 2011	RCT<br>3 arms: Intervention (13), CI (13), NTC (13)<br>N=39<br>One HEI, Australia	Selective/indicated<br><br>Undergraduate psychology and health sciences students<br>77% female, M age 19.6 yrs<br>Low-to-moderate levels of psychological distress (K-10)	*MoodGym*: internet-delivered CBT program<br><br>Semi-guided	Five modules accessed during three 60-minute sessions over three weeks	Researcher-monitored laboratory	DASS-21<br>ATQ<br>K10<br>CBT-Lit<br>OSSS	ITT
Kanekar et al 2010*	RCT<br>2 arms: Intervention, AC<br>N=60; 39 analysed<br>One HEI, USA	Universal<br><br>Postgraduate students<br>34 male, M age 24.6yrs<br>Full-time international students of Indian nationality	Internet-delivered intervention: sessions focused on education about social support, hardiness and acculturation<br><br>Semi-guided	Three sessions completed over eight weeks	Own location	K-10 (analysed as K-6)*<br>ISEL<br>AIRS<br>Hardiness scale	Completers
Kenardy et al 2003	RCT<br>2 arms: Intervention (43), WLC (40)<br>N=83<br>One HEI, Australia	Selective/indicated<br><br>Undergraduate psychology students<br>65 females, M age 19.9yrs<br>Elevated anxiety sensitivity levels (ASI)	*Online Anxiety Prevention Program*: psycho-educational and CBT-based internet-delivered intervention<br><br>Self-administered	Six sessions over six weeks<br><br>Recommended to spend 5-7 days working on each session	Own location	ASI<br>CES-D<br>BSQ<br>CCQ-M<br>ACQ	Completers
Lintvedt et al 2011	RCT<br>2 arms: Intervention (81), WLC (82)<br>N=163<br>Two HEIs, Norway	Selective/indicated<br><br>Undergraduate and postgraduate students<br>125 females, M age 28.2yrs<br>Elevated levels of psychological distress (K-10)	*MoodGym*: internet-delivered CBT program + *BluePages*: informational website about depression<br><br>Unsure about level of human support	Five *MoodGym* modules made available on sequential weekly basis. Trial period was eight weeks. Participants given manuals detailing which parts of intervention to access weekly.	Own location	CES-D<br>ATQ<br>TDL	ITT (Completers' analysis reported separately)

Orbach et al 2007	RCT<br>2 arms: Intervention (47), AC (43)<br>N=90<br>One HEI, UK	Selective/indicated<br><br>Undergraduate and postgraduate students<br>58 female, M age 24.2yrs<br>Not receiving treatment for test anxiety<br>Self-reported text anxiety	Internet delivered intervention for test anxiety; based on CBT<br><br>Self-administered	Six modules; suggested dosage 30 minutes per week (but not mandatory)	Own location	TAI<br>AHQ<br>EPSI<br>GSES<br>STAI-S	Completers
Radhu et al 2012	RCT<br>2 arms: Intervention (28), WLC (30)<br>N=58; 47 analysed<br>One HEI, Canada	Selective/indicated<br><br>Undergraduate psychology students<br>34 female, M age 22.6yrs<br>High levels of perfectionism (PCI)	Internet-delivered CBT for perfectionism (same as Arpin-Cribbie 2012)<br><br>Self-administered	13 modules over 12 weeks; could access anytime	Own location	CES-D<br>ATQ<br>BAI<br>ASI<br>PSS<br>MPS-F<br>MPS-HF<br>PCI<br>APSR	Completers
Rose et al 2013	RCT<br>2 arms:<br>Intervention (34), AC (32)<br>N=66; 59 analysed<br>One HEI, USA	Selective/indicated<br><br>Postgraduate students, mostly from Management and Law courses<br>33 females, M age 27.3yrs<br>Elevated stress (PSS)	*SMART-OP*: CBT-baeed stress management and resilience training program.<br>Delivered via computer program<br><br>6 sessions over 6 weeks + homework between sessions + reminder emails<br><br>Semi-guided	Six sessions over six weeks; accessed once a week + homework activities between sessions	Researcher monitored laboratory	PSS<br>SPCS<br>Trier Social Stress Test<br>Biological markers: a-amylase (saliva), heart rate + variability (ECG), skin conductance, systolic and diastolic blood pressure	Completers
Sethi et al 2010	RCT<br>4 arms:<br>Intervention (9), CI 1 (10), CI 2 (9), NTC (10)<br>N=38<br>One HEI, Australia	Selective/indicated<br><br>Undergraduate psychology students<br>25 females, M age 19.47yrs<br>Low-to-moderate depression and/or anxiety (DASS-21)	*MoodGym*: internet-delivered CBT program<br><br>Semi-guided	Five modules accessed during three sessions over three weeks	Researcher-monitored laboratory	DASS-21<br>K10<br>ATQ	ITT

Taitz 2011	RCT<br>2 arms: Intervention (86), NTC (89)<br>N=175<br>One HEI, USA	Universal<br><br>Undergraduate students<br>150 female, M age 20.7yrs<br>≥18 yrs, no lucid dreaming experience	Lucid dreaming instruction taught via the internet<br><br>Self-administered	Website accessed once a week over four week period	Own location	BDI<br>LOC scale<br>Lucid Dream Recall + Control questionnaire<br>Dream recall questionnaire	Uncertain

Abbreviations: RCT – randomised controlled trial; AC – active control; CI – comparison intervention; WLC – wait-list control; NTC – no treatment control; ITT – intention-to-treat analysis; HEI – higher education institution; CBT – cognitive-behaviour therapy.

Measures: ACQ - Agoraphobia Cognitions Questionnaire; AHQ – Anxiety Hierarchy Questionnaire; AIRS – American International Relations Scale; APSR - Almost Perfect Scale–Revised; ASI - Anxiety Sensitivity Index; ATQ - Automatic Thoughts Questionnaire; BAI - Beck Anxiety Inventory; BDI - Beck Depression Inventory ; BFNE – Fear of Negative Evaluation scale; BSQ - Body Sensations Questionnaire; CAS - College Adjustment Scales; CBT-Lit - Cognitive Behavioural Therapy Literacy Scale; CCQ-M - Catastrophic Cognitions Questionnaire-Modified ; CES-D - Centre for Epidemiologic Studies–Depressed Mood Scale; CPQ-CC - Communication Patterns Questionnaire - Constructive Communication Subscale; CSI - Couples Satisfaction Index; CTS-2 - Revised Conflict Tactics Scale; DASS-21 - Depression, Anxiety and Stress Scale – 21 item version; EPSI – Exam Problem-Solving Inventory; FFMQ – Five Facet Mindfulness Questionnaire; FPSQ – Fear of Public Speaking Questionnaire; GSES - General Self-Efficacy Scale —exam related; HPLP-II - Health-Promoting Lifestyle Profile II; ISEL – Interpersonal Support Evaluation list; K-10 - Kessler Psychological Distress Scale; LOC scale - Internal/External Locus of Control Scale; MPS-FM-CM - Multidimensional Perfectionism Scale - Concern over Mistakes subscale ; MPS-HF - Multidimensional Perfectionism Scale; MS – Maladjustment scale; Online Social Support Scale (OSSS); PANAS - Positive and Negative Affect Schedule; PCI - Perfectionism Cognitions; PRQC - Perceived Relationship Quality Components Inventory; PSS - Perceived Stress Scale; SAD – Social Avoidance and Distress Scale; SPCS – Stress and Perception of Control Scale; SSPS – Self-Statements During Public Speaking; STAI-S - State Trait Anxiety Inventory – State; TAI - Test Anxiety Inventory; TDL – Treatment Depression Literacy.
