# Supplementary material for: Computer-Delivered and Web-Based Interventions to Improve Depression, Anxiety, and Psychological Well-Being of University Students: A Systematic Review and Meta-Analysis
Source: J Med Internet Res. 2014 May 16;16(5):e130. doi: 10.2196/jmir.3142 (PMC4051748; doi:10.2196/jmir.3142)
Supplement: Supplementary file 5 [file jmir_v16i5e130_app5.pdf]

Multimedia Appendix 4. Non-skewed data which could not be incorporated into meta-analyses due to being sole study for specific outcomes of interest.

Comparison: Website-based or computer-delivered intervention compared to inactive control

Psychological distress		N	Mean (SD)
Sethi 2010	Intervention	9	17.8 (5.9)
	No treatment control	10	31.1 (3.3)

Comparison: Website-based or computer-delivered intervention compared to active control

Stress			
Rose 2013	Intervention	30	11.93 (3.85)
	Active control	29	14.48 (5.21)

Comparison: Website-based or computer-delivered intervention compared to comparison intervention

Anxiety		N	Mean (SD)
Sethi 2010	Intervention	9	8.6 (4.1)
	Comparison	10	8.0 (3.2)
Depression			
Sethi 2010	Intervention	9	15.7 (4.2)
	Comparison	10	7.2 (3.1)
Psychological distress			
Sethi 2010	Intervention	9	17.8 (5.9)
	Comparison	10	13.8 (2.04)
